# Factors affecting fisher decisions: The case of the inshore fishery for European sea bass (*Dicentrarchus labrax*)

**DOI:** 10.1371/journal.pone.0266170

**Published:** 2022-03-31

**Authors:** Joseph W. Watson, Angela Muench, Kieran Hyder, Richard Sibly

**Affiliations:** 1 School of Biological Sciences, University of Reading, Reading, Berkshire, United Kingdom; 2 Centre for Environment, Fisheries & Aquaculture Science, Lowestoft Laboratory, Lowestoft, Suffolk, United Kingdom; 3 School of Environmental Sciences, University of East Anglia, Norwich, Norfolk, United Kingdom; Swedish University of Agricultural Science, SWEDEN

## Abstract

Fishery management relies on forecasts of fish abundance over time and space, on scales of months and kilometres. While much research has focussed on the drivers of fish populations, there has been less investigation of the decisions made day-to-day by fishers and their subsequent impact on fishing pressure. Studies that focus on the fisher decisions of smaller vessels may be particularly important due to the prevalence of smaller vessels in many fisheries and their potential vulnerability to bad weather and economic change. Here we outline a methodology with which to identify the factors affecting fisher decisions and success as well as quantifying their effects. We analyse first the decision of when to leave port, and then the success of the fishing trip. Fisher behaviour is here analysed in terms of the decisions taken by fishers in response to bio-physical and socio-economic changes and to illustrate our method, we describe its application to the under 10-meter fleet targeting sea bass in the UK. We document the effects of wave height and show with increasing wave height fewer vessels left port to go fishing. The decision to leave port was only substantially affected by time of high tide at one of the four ports investigated. We measured the success of fishing trips by the landings of sea bass (kg) per metre of vessel length. Fishing success was lower when wave height was greater and when fish price had increased relative to the previous trip. Fuel price was unimportant, but a large proportion of the variation in success was explained by variation between individual vessels, presumably due to variation in skipper ability or technical restrictions due to vessel characteristics. The results are discussed in the context of management of sea bass and other small-scale inshore fisheries.

## 1. Introduction

The global state of fish stocks is a cause for concern, and there is a need for increasingly effective fisheries management [1]. An area of management that has received less research attention is the human element of fisher behaviour [2]. As it is ultimately the fishers and not the fish that managers can directly influence, it is critical for successful management that fisher behaviour is taken into consideration [3, 4].

Fisher behaviour is analysed here in terms of the decisions taken by fishers in response to bio-physical and socio-economic changes, as recently reviewed by Andrews, Pittman and Armitage [5]. Many of the studies [3, 4, 6–8] have sought to establish how management decisions affect the dynamics and distribution of fisher decisions and the subsequent pressure on the fishery. The drivers of fisher decisions are often complex and interlinked but can be coarsely categorised into environmental, economic, and legislative. Economic factors include fluctuations in fuel or fish price and their interaction through the market can have profound impacts on fisheries [9], as can be seen for example in the changes of demand and supply during the COVID-19 pandemic [10]. Profitability is often a balance of both environmental and economic factors, but is also affected by legislation. Legislation can be broad and applied in a variety of ways, including restrictions on quota or fishing gears in addition to spatial or temporal closures, all of which can have major influence on fisher decisions [11, 12]. Environmental drivers, such as weather and climate change including increasing storminess, have already been shown to affect fishing decisions and subsequent fishing pressure in some cases [13–15]. Fisheries are likely to be exposed to a combination of pressures and the relevance and magnitude of fisher decisions may vary between different fisheries. Understanding of these relationships is increasingly recognised as an important component of fisheries management [2].

The fishing method used to target catch is one obvious aspect of a fishery that will affect how different environmental, economic, and legislative pressures impact fisher decisions. Globally, fishing methods are extremely diverse, but often involve the use of a dedicated fishing vessel. These fishing vessels can range from small canoes up to factory trawler ships, and the decisions of fishers operating on vessels of different sizes may be affected by different predictors [16, 17]. Research into fisher behaviour is necessarily dependant on the data available. To gain detailed insight into spatial fishing pressure, studies that focus on European vessels longer than 12-meters can make use of data from Vessel Monitoring Systems (VMS) or Automatic Identification System (AIS) for vessels over 300 gross tonnes engaged in international voyages. However, for smaller vessels AIS is limited as it is voluntary [18] and VMS is not required in European waters for these vessels. Smaller vessels are also potentially more vulnerable to environmental change [13, 19] and importantly, despite their small size, small vessels make up a large percentage of global fisheries with 82% of recorded motorized fishing vessel lengths being less than 12 meters [20]. It is therefore important to consider, for both small and larger fishing vessels, all available information in trying to understand fisher decisions and their impact on fishing pressure.

In this study, we focus on smaller vessels and use as a case study the UK under 10-meter fleet catching European sea bass (*Dicentrachus labrax*, *Moronidae*) in the North Sea, English Channel, Celtic Sea, Bristol Channel, and Irish Sea (Northern Stock, ICES 4b&c, 7a,d-h). Sea bass is a large, high value, slow growing and late maturing fish that until 2015 was not subject to catch restrictions. In the past decade, the northern stock size fell rapidly, which was attributed to a combination of poor year classes and fishing mortality [21]. The decline led to the implementation of emergency management measures in 2015 [21], and, since 2020, UK vessels have been limited to targeting sea bass with hook and line, and bycatch limits for fixed gill nets, seine nets, and trawls [22]. Sea bass continues to be an important species of the UK under 10-meter fleet as it is a high value species that can be harvested close to shore [23].

In an attempt to gain insight into the complex decisions made by fishers using smaller fishing vessels, we analyse first the decision of when to leave port, and then the success of the fishing trip. To demonstrate our approach, we assess the impact of environmental and socio-economic drivers on under 10-meter fishery for sea bass. We collate data from a number of different sources which we use to predict when fishing trips occur and their success as measured by landings. Based on a linear regression approach, we assess the importance of different factors driving decisions to leave port and fishing success. The results are discussed in the context of management of sea bass and other small-scale inshore fisheries.

## 2. Methods

Our analysis has two components:

The decision to leave the port analysed here by a logistic model we term *Leave port*.

The success of the fishing trip analysed here by a linear regression model we term *Fisher success*.

Models are created for each of these processes independently. This is done by identifying possible predictors of fisher decisions and then attempting to obtain relevant data. The approach for this will vary extensively between fisheries, but to give an idea of how it can be done in practice, we illustrate our method below with a case study of the under 10m fleet of UK northern stock sea bass fishery.

### 2.1 Identifying possible predictors

Both fuel price and weather have been identified as explanatory variables in other fisher behaviour studies [9, 14]. Time of high water is a further environmental driver that is likely to affect the decision to leave port due to *a priori* understanding of logistical issues of low tides (e.g., navigating shallow water and ability to leave tidal moorings). To our knowledge daily tide cycles have not been included in fisher behaviour analyses until now, though Sharples *et al*. [24] studied Celtic sea fishing activity in response to spring and neap tides and Poisson *et al*. [25] assessed monthly tidal influence for a Réunion Island longline fishery. Inclusion of further possible fisheries behaviour predictors is necessarily constrained by the availability of data. In the case of the UK northern stock sea bass fishery, the best data source available to record when vessels leave port and their success are the Marine Management Organisation (MMO) logbooks, whose contents are described below. MMO logbooks contain information beyond a simple yes/no answer for leaving port, however we do not have any information on the reasoning when vessels have remained in port, so we cannot assess this in our case study leave port analysis. It was possible however to supplement logbook data with data from other sources, here in our case study we were able to obtain data on time of high water, wave height and fuel price (details shown below).

For the fishing success analysis, we defined the dependent variable, termed fisher success, as landings per metre of vessel length in order to standardise the outcome of fishing trips for differences in vessel size. For this analysis, we were able to use all the data in the MMO logbooks (in addition to our extra data sources) as predictors of fisher success, namely *wave height*, *tide*, *change in fish price*, *fuel price*, *month*, and *year*. One key recording from the MMO logbooks is fish price, however rather than use fish price directly we used the *change in fish price* since the previous fishing trip to account for potential changes in revenue and therewith profit [26]. *Wave height*, to the nearest metre, was entered as a factor to capture any non-linear effects. Individual *vessel ID* and *port name* were entered as fixed effects to reveal associated unobserved effects of vessel and location. Yearly fixed effects capture the annual changes in fisheries legislation which might restrict harvest success. Vessel fixed effects captures skipper ability as well as capacity or technical restrictions due to vessel characteristics. We chose predictors from the logbook and other data sources on the assumption that they are likely predictors of fishing success and are of relevance to legislation and future management decisions [22, 27–30].

### 2.2 Obtaining data

**Fig 1:**
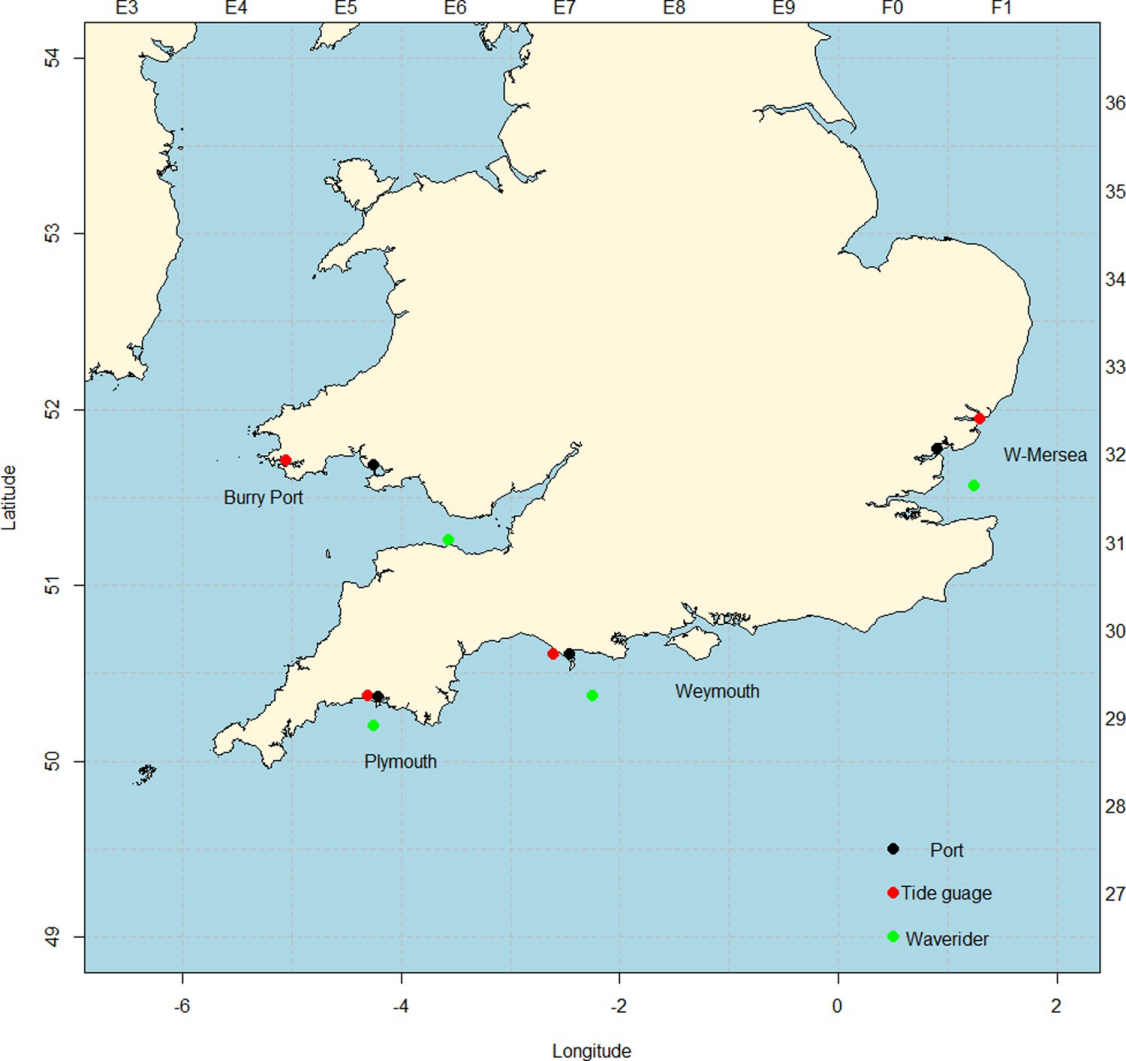
Map of study ports, and instruments from which data was taken. Black dots indicate the study port locations. Red dots and Green dots show the approximate location of tide gauges and wave rider buoys respectively (The map was produced in R version 3.6.1 [32] with the package mapplots [31]).

In the case of the UK northern stock sea bass fishery, the main data source of when vessels leave port and their success are the MMO logbooks which incorporates sales notes for the under 10-meter vessels in similar format. In addition to recording the days on which named vessels left named ports, the logbooks record the weight of fish caught. These records were supplemented by data on wave height, time of high tide and fuel price. We analysed individual trip data for the years 2014–2018 for vessels of up to 10 meters in length for four study ports: Burry Port; Plymouth; West Mersea; and Weymouth (Fig 1, Tables 1 and 2). The chosen ports represent the fishery spatially, each being chosen on the basis that it had the highest annual value landings of sea bass within its region (logbook data 2014–2018). Each study port had a fleet consisting of vessels with lengths over and under 10-meter, but sea bass fishing was more valuable to the under 10-meter vessels in all four ports (Table 1). We define vessels that are sea bass-targeting/impacting as those that recorded more than 10 trips with more than 10% landings by weight of sea bass. The resulting dataset contains 8,815 fishing trips between 2014–2018 (Table 2). The study ports differed in the number, size, and engine power of the vessels in their fleets, and the total landings of sea bass varied between ports with Weymouth catching the most sea bass (Table 2). Fishing gear also varied with more sea bass caught using hook and line than other fishing methods in all our study ports except West Mersea, where gill nets were favoured (Table 2).

**Table 1:** Descriptive statistics of the chosen ports from MMO landings data 2014–2018 (< 10 or > 10 indicated under & over 10-meter fleet respectively).

Port name	Total landings (t)		Bass Landings (t)		Bass % of total value of catch		Location in England & Wales
	<10	>10	<10	>10	<10	>10	
Burry Port	247	-	129	-	87	-	West
Plymouth	4207	47320	137	44.6	15	0.63	South-West
West Mersea	580	68	74	0.4	44	1.66	East
Weymouth	1891	6356	254	0.6	44	0.03	South

**Table 2:** Descriptive statistics of chosen vessels from MMO logbook scheme.

Port name	no. vessels	no. trips	Vessel Length (m)		Vessel Power (hp)		Landings (t)	% caught by gear			
			r.	m.	r.	m.		GN	HL	TRP	TRW
Burry Port	42	2416	4.5–10	5.7	15–170	58	97	36	64	0	0
Plymouth	46	3342	4.0–10	6.4	9–216	53	113	38	62	0.2	0.2
West Mersea	14	679	4.6–10	7.7	4–157	54	64	96	0.2	0.1	3.8
Weymouth	40	2378	4.3–10	7.5	4–158	103	200	11	89	0.1	0.8

*no*. *vessels* = number of vessels per port, *no*. *trips* = total number of fishing trips for all vessels in each port, *r*. = range, *m*. = mean, *GN* = Gill net, *HL* = Hook and line, *TRP* = Traps/Pots, *TRW* = Trawls.

We collated several environmental and socio-economic parameters for use in the analysis. Out of possible weather variables, we use *Wave height* to represent sea conditions due to availability of data and its convenience as a combination of wind speed, and direction. *Wave height* was taken from the UK strategic wave monitoring network WaveNet(Source: https://www.cefas.co.uk/data-and-publications/wavenet/ [last access: 02/02/2021]). The closest Waverider buoys to our focus ports (Fig 1) were used to calculate daily average *wave height*. The buoys are not always stationed directly outside our study ports but gave an adequate representation of the daily sea state for our purpose. To calculate *time of high tide*, we first obtained data on tidal movements, from the British Oceanographic Data Centre’s (BODC) tide gauge archive (Source: https://www.bodc.ac.uk/data/hosted_data_systems/sea_level/uk_tide_gauge_network/ [last access: 02/02/2021]). The time of tide measurements were rounded to the nearest hour, we then checked in the first 12 hours (00:01–11:59) of each 24-hour period for the highest water and corresponding time, taking this as the first high tide of the day. We only used the first high tide time in our analysis as first and second tide times are closely correlated. Tide gauges are not all stationed directly outside our study ports (Fig 1) but should give an adequate representative of tide state for our purpose.

To calculate *price change*, we extracted the mean daily price of sea bass for each port (£.kg^-1^) from the MMO logbooks. We then subtracted the price received by the vessel on its previous trip. The resulting *price change* is either a positive or negative value indicating a price rise or drop, respectively. Data from February and March 2016–2018 were excluded because there was a ban on fishing for sea bass in these months [27–29]. To estimate *fuel price*, because daily fuel price at each port was not recorded, we used monthly red diesel prices (Source: https://www.gov.uk/government/statistical-data-sets/oil-and-petroleum-products-monthly-statistics [last access: 02/02/2021]) on the assumption that fishing vessels used untaxed diesel or other fuels (e.g., regular pump petrol) correlated to these prices.

All statistical analyses were carried out in R (version 3.6.1 [32]). Final estimations were derived by backwards stepwise regression (StepAICc MASS—[32]) and a Likelihood ratio test (Step Stats [33]). We checked for collinearity in model predictors using correlation matrixes and analysing variance inflation factor (VIF) scores.

## 3. Results

### 3.1 Leave port model

To assess the effects of factors affecting the decision of a fishing vessel to leave port, we used a binary logistic regression. The dependent variable was whether or not the vessel left port to go fishing, and the predictors were: *Wave height*, entered as a continuous variable, and *port* and *time of high tide*, entered as fixed factors. *Fuel price* was not included in the final model for reasons given in the discussion below. To identify any regional differences between ports, we included interaction terms port*wave height and port*time of high tide. It is not possible to use other information from the MMO logbook as predictors of whether a vessel leaves port as we only have data when vessels do leave, with unknowns when they do not.

**Fig 2:**
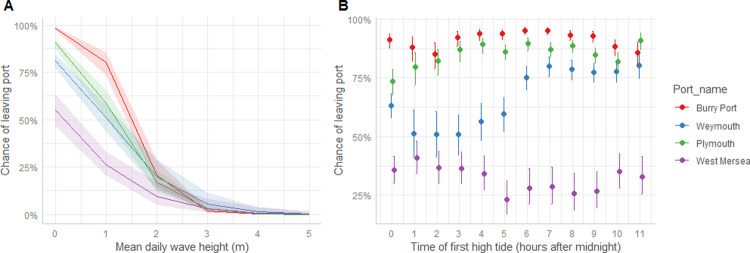
Predictors of whether a vessel will leave port from the binary logistic regression. A) mean significant wave height, B) time of first high tide. Bars and bands indicate confidence intervals. For both figures, colours are used to distinguish between ports where Red = Burry port, Blue = Plymouth, Green = West Mersea and Purple = Weymouth.

All predictors entered into the leave port model significantly affected vessel decision to leave port and go fishing (Table 3). In calm conditions (wave height less than a meter) most vessels left port, except from West Mersea where the proportion leaving port was lower (Fig 2A). As wave height increased, fewer vessels left port to go fishing. Fewer than 25% left port when wave height exceeded 2 meters, and very few when wave height was over 3 meters (Fig 2A). The effect of time of high tide is shown in Fig 2B. The decision to leave port was little affected by time of high water except at Weymouth, where there was a distinct preference for later tides, between 6 a.m. and 11 a.m. (Fig 2B). We show a confusion matrix (Table 4) from which we can report model scores of; Sensitivity = 91%, Specificity = 50%, Precision = 84% and Accuracy = 80%.

**Table 3:** Analysis of deviance table for the *Leave Port* model. The dependent variable was whether or not a vessel left port to go fishing.

Predictor	Df	Deviance	Resid. Df	Resid. Dev	Pr(>Chi)
NULL			13286	15329	
Time of high tide (HT)	11	183	13275	15146	***
Port name (PN)	3	1307	13272	13839	***
Wave height (WH)	1	1826	13271	12014	***
HTxPN	33	210	13238	11803	***
WHxPN	3	82	13235	11721	***

*** < 0.0005.

VIF range 1.09–1.46.

Cragg-Uhler pseudo-R2 = 0.35 for 51 df.

**Table 4:** Confusion matrix for the *Leave Port* model.

		Predicted Value	
Actual Value		FALSE	TRUE
	0	1755	1748
	1	926	8858

### 3.2 Fisher success model

**Fig 3:**
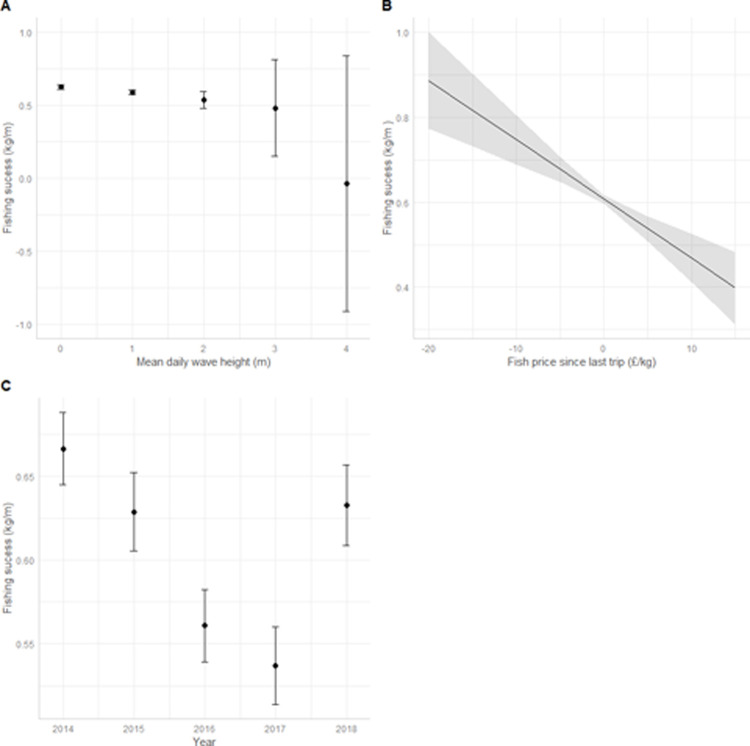
Effects of predictors on fishing success, from the regression analysis (Eqn. 2). A) Effect of mean daily wave height; B) Change in fish price from last trip; C) year the fishing trip took place. Bars and bands indicate confidence intervals.

Fisher success is defined in this study as the natural logarithm of the landings of sea bass (kg) per metre of vessel length. We used a general linear model to assess the effects on fishing success of environmental and socio-economic variables. *Wave height*, *vessel identity* and *year* were entered as fixed factors and *change in fish price* was entered as a continuous variable. *Fuel price*, *time of high tide* and *month* were not included in the final model for reasons given in the discussion below. *Port* was also not included in this model as a result of a backwards stepwise regression used for model selection. All predictors entered into the final regression model significantly affected the success of the fishing trip (Table 5). Fishing success was lower when wave height was greater (Fig 3A) and when fish price had increased relative to the previous trip (Fig 3B). Finally, a large proportion of the total sum of squares was explained by factors associated with individual vessel (*Vessel ID*, Table 5).

**Table 5:** Analysis of variance table for the *Fisher Success* model. The dependent variable was landed weight of sea bass per meter of vessel.

Predictor	Df	Sum Sq	Mean Sq	F value	Pr(>F)
Wave height (As factor)	4	11.09	2.7736	13.990	***
Change in fish price	1	4.97	4.9685	25.061	***
Year	4	16.83	4.2073	21.221	***
Vessel ID	138	693.20	5.0232	25.336	***
Residuals	8667	1718.32	0.1983		

*** < 0.0005.

VIF range 1.01–2.69.

R^2^ = 0.28.

## 4. Discussion

In this study, we demonstrate a general fisher behaviour modelling approach which analyses separately the decision to leave a fishing port and the impact of decisions on fishing trip success. When applying our method, it is important to note that each fishery will be unique in the data available and the predictors that significantly affect fisher decisions. We demonstrate our approach with a case study investigating the decisions of fishers in under 10-meter sea bass fishing vessels at four UK representative ports, aiming to identify how decisions are affected by socio-economic and environmental factors.

In both analyses we discarded some predictors because their estimated effects are *a priori* implausible, so including them could distort the analyses. Results including those variables are shown in S1 and S2 Figs in S1 File. For both models we discarded *fuel price*, because increased fuel price was found to correlate with more trips and with more successful trips, which seem *a priori* implausible. Results including *fuel price* are shown in S1 and S2 Figs in S1 File. For the Fisher Success model, we discarded *time of high tide* and *Month* from our analysis as we lack a sensible explanation of their effects. Tide time did not show consistent patterns hour-to-hour, unlike the Leave port model (compare S1A Fig in S1 File and Fig 2B). Including the effect of month on fishing success suggests that December is the most profitable month to fish (S2C Fig in S1 File). This is unlikely to be a reliable result to include in the wider analysis as there are fewer fishing trips that occur in December compared to during peak fishing, from April to October [34]. Hence, it is likely that the high profitability of December fishing is an artifact of incidental sea bass landings.

Our principal findings are that almost all vessels left port when wave height was below a meter, but less than a quarter when wave height exceeded 2 meters, and those that did then leave caught less. Our finding that in rougher weather fewer vessels leave the port to go fishing (Fig 2A) is in line with other studies of fisher behaviour [13, 14]. Due to their small physical size the small vessels that make up the under 10-meter fleet have potential to be particularly vulnerable to rough weather. The port with the smallest mean vessel size is Bury Port (Table 2) and this is the port seemingly most impacted by wave height (S1 Table in S1 File), though note its distance from its Waverider buoy (Fig 1).

Decisions to leave port were also affected by the time of high water. We describe the variation between ports, and present quantitative estimates of all effects. To our knowledge, there is only limited incorporation of environmental predictors other than weather variables in studies of fisher behaviours. Daily tidal state has not been included in any fisher behaviour study that we are aware of, though studies by Sharples *et al*. [24] and Poisson *et al*. [25] show results of fishers reacting differently throughout the monthly tide cycles depending on their target species. In our study, the vessels we have defined as targeting/impacting sea bass (see section 2.2 obtaining data) appear to have fishing decisions to leave port affected by the time of high water. However, the effects of daily tide cycle did differ between ports (Fig 2B). Depth of water may limit the ability to leave or return to a tidal mooring, so leaving on an early tide may allow a fisher to stay out at sea and fish through two tide cycles rather than be limited to one. Early tides may also allow fishers more sociable hours and/or to fish in daylight. The preference for certain tide times could also be due to a perceived increased chance of catching sea bass and/or due to logistical preferences. Fishers may be attracted to certain tide times as changes in current velocity could carry the scent of bait further and also have a direct impact on feeding behaviour of fish [35]. Empirical studies of these effects are rare [35], but grey literature in fishing magazines suggests sea bass have greater feeding activity during times of tidal movement, making them potentially profitable times to go fishing. A final consideration is the different effect tide can have on different fishing gears [24]. West Mersea was shown to have a different response to tidal effects than the other ports (Fig 2B), contributing to this could be the prevalence there of using gill nets, which is different to the majority of vessels in other ports that used hook and line (Table 2).

The success of fishing trips, as measured by landed weight of sea bass per metre of vessel length, was generally greater in calmer seas (Fig 3A). Fewer vessels go fishing in rougher weather (Fig 2A) and the success of the vessels that do fish is reduced (Fig 3A), reasons could include: not being able to fish the best/preferred fishing marks [36], being unable to deploy as much fishing gear (e.g., number of hooks), or because time spent at sea is reduced. Given the major effect of wave height on decision to leave port and fisher success, any change in storminess due to climate change [13] could have implications for sea bass fishing pressure. Increased future storminess would result in more days when fishing is not possible and could result in significant changes to the spatial and temporal distribution of fishing pressure. In addition to changes in fisher behaviour, climate change has the potential to effect the distributions and reproductive biology of the sea bass that they target [37, 38]. This combination of climate change effects on both sea bass and the fishers that target them could have compounding impact on the dynamics and distribution of future sea bass fishing pressure.

The success of fishing trips was also greater when the change in fish price, compared with the previous trip, was lower (Fig 3B). Amongst other factors affecting success of catch, bad weather may help explain this because prices are inflated when fewer fish are brought to market due to adverse fishing conditions such as bad weather [39]. Although the increase in storminess may impact when and where sea bass are landed, the economic outcome may have a limited net change. Fishing success varied between years, being greatest in 2014 (Fig 3C). Fishing success varied substantially between vessels (Table 5), this is likely due to a variety of reasons including the effects of seasonality on individual trips but also variation in skipper experience and risk perception [8], sometimes termed the skipper effect [40]. Although beyond the scope of this study, further insight into the skipper effect is often gained from semi-structured interviews and other survey techniques [14, 41, 42].

We cover the top port per region for sea bass landings in the UK 2014–2018, and our logbook data covers at least 75% of the total trips per port (Tables 1 and 2). However, since our analysis shows ports react differently to environmental and socio-economic predictors, it is likely that other UK ports not included in this study may also differ. Furthermore, we know there is a 25 kg exemption from sales notes for landed sea bass, meaning that some landings of sea bass are unreported [43] and some discard mortality that cannot be captured, resulting in potential underestimation of fishing pressure and mortality. There is also potential scope to add more predictors as new data becomes available, with increasing number of variables it may be necessary to use more complex model selection methods (i.e., Lasso [44]). The additions of more data as they come available may also help explain the impact of fuel price and other variables which were omitted from the current analysis (see earlier in the discussion). Nevertheless, we believe the study presented here is a good starting point to indicate some of the mechanisms of fishing pressure responses between ports.

These findings have implications for the management of sea bass. Management is through technical measures that include catch limits (monthly, bimonthly, annual), closed seasons to protect spawning aggregations, and minimum size [22]. Increases of extreme weather events especially during the key fishing seasons may impact on the ability of under 10-meter inshore vessels to land catch limits within the allowed time periods. As these are time bound and there is no carryover, this will impact the potential revenue generated and therewith the profit. It may also be the case that as the stock expands northwards, due to warming sea temperatures, any seasonal closures may not protect spawning aggregations in all areas.

To use our method as an adaptive management tool, for our case study and other fisheries, it would be useful to consider the spatial aspect of fisher behaviour. Spatial data is not necessary for estimating total pressure on the stock, but it is important when investigating the spatial concentration of fishing effort and the pressure of fishing near protected areas [45]. A promising line of future work would be to incorporate our fisher behaviour findings into an individual based model (IBM). IBMs use a bottom-up approach and simulate a population of discreet individuals where a combination of individual state and environmental variables change individual behaviour [46]. IBMs have been used in fisheries research to study fish populations [47–51], but have also been used to study fisher behaviour [52–56]. We suggest that incorporation of the fishing behaviour relationships we have found into a suitable IBM could be a useful management tool.

## 5. Conclusions

The primary findings from this study relate to the effect of wave height on the under 10-meter inshore vessels that target or impact sea bass around the UK. We found that fewer vessels left port during rough weather to go fishing and vessels that did were less successful. Fishers were also more successful when fish price had decreased relative to the previous trip, due to supply/demand. The decision to leave port was only substantially affected by time of high tide at one of the four ports investigated. Fuel price was unimportant, but a large proportion of the variation in success was explained by variation between individual vessels, presumably due to variation in skipper ability or technical restrictions due to vessel characteristics. The findings from this study have implications for the management of sea bass fishing pressure as any increases of extreme weather events during the key fishing seasons may affect the ability of under 10-meter inshore vessels to land catch limits within the allowed time periods. As these are time bound and there is no carryover, this will impact the potential revenue generated and profit. We hope the methodology employed here will prove useful in future studies seeking to identify and quantify the effects of factors affecting fisher decisions and success.

## Supporting information

S1 File (DOCX)
