# Supplementary material for: Factors affecting fisher decisions: The case of the inshore fishery for European sea bass (Dicentrarchus labrax)
Source: PLoS One. 2022 Mar 31;17(3):e0266170. doi: 10.1371/journal.pone.0266170 (PMC8970468; doi:10.1371/journal.pone.0266170)
Supplement: S1 File — (DOCX) [file pone.0266170.s001.docx]

**Supplementary**

**Table S1.** Coefficients for leaveport model Eqn. 1

| **Coefficients:** | **Estimates** | **Std. Error** | **Pr(>\|z\|)** |
| --- | --- | --- | --- |
| (Intercept) | 4.12009 | 0.21632 | *** |
| Time_F1 | -0.35324 | 0.3136 |  |
| Time_F2 | -0.59732 | 0.28589 | * |
| Time_F3 | 0.10743 | 0.28563 |  |
| Time_F4 | 0.36582 | 0.2728 |  |
| Time_F5 | 0.35582 | 0.2538 |  |
| Time_F6 | 0.63745 | 0.25598 | * |
| Time_F7 | 0.60632 | 0.25243 | * |
| Time_F8 | 0.25861 | 0.24568 |  |
| Time_F9 | 0.2166 | 0.24688 |  |
| Time_F10 | -0.338 | 0.24612 |  |
| Time_F11 | -0.55768 | 0.2883 | . |
| Port_namePlymouth | -1.867 | 0.2675 | *** |
| Port_nameWest Mersea | -3.91104 | 0.27867 | *** |
| Port_nameWeymouth | -2.6563 | 0.24813 | *** |
| mean_hs | -2.72693 | 0.10519 | *** |
| Time_F1:Port_namePlymouth | 0.70653 | 0.41023 | . |
| Time_F2:Port_namePlymouth | 1.10812 | 0.37294 | ** |
| Time_F3:Port_namePlymouth | 0.76895 | 0.3771 | * |
| Time_F4:Port_namePlymouth | 0.7316 | 0.35494 | * |
| Time_F5:Port_namePlymouth | 0.45929 | 0.32607 |  |
| Time_F6:Port_namePlymouth | 0.48955 | 0.33135 |  |
| Time_F7:Port_namePlymouth | 0.29396 | 0.32433 |  |
| Time_F8:Port_namePlymouth | 0.77894 | 0.31797 | * |
| Time_F9:Port_namePlymouth | 0.48716 | 0.31605 |  |
| Time_F10:Port_namePlymouth | 0.83087 | 0.32234 | ** |
| Time_F11:Port_namePlymouth | 1.85447 | 0.39789 | *** |
| Time_F1:Port_nameWest Mersea | 0.575 | 0.36751 |  |
| Time_F2:Port_nameWest Mersea | 0.64275 | 0.347 | . |
| Time_F3:Port_nameWest Mersea | -0.06729 | 0.34582 |  |
| Time_F4:Port_nameWest Mersea | -0.43089 | 0.34305 |  |
| Time_F5:Port_nameWest Mersea | -0.95588 | 0.351 | ** |
| Time_F6:Port_nameWest Mersea | -0.98164 | 0.3457 | ** |
| Time_F7:Port_nameWest Mersea | -0.93353 | 0.34348 | ** |
| Time_F8:Port_nameWest Mersea | -0.72119 | 0.34796 | * |
| Time_F9:Port_nameWest Mersea | -0.63316 | 0.34232 | . |
| Time_F10:Port_nameWest Mersea | 0.31037 | 0.32386 |  |
| Time_F11:Port_nameWest Mersea | 0.44027 | 0.36608 |  |
| Time_F1:Port_nameWeymouth | -0.13857 | 0.39052 |  |
| Time_F2:Port_nameWeymouth | 0.09285 | 0.36526 |  |
| Time_F3:Port_nameWeymouth | -0.61662 | 0.35394 | . |
| Time_F4:Port_nameWeymouth | -0.64804 | 0.33936 | . |
| Time_F5:Port_nameWeymouth | -0.50917 | 0.31648 |  |
| Time_F6:Port_nameWeymouth | -0.0722 | 0.31454 |  |
| Time_F7:Port_nameWeymouth | 0.23878 | 0.30617 |  |
| Time_F8:Port_nameWeymouth | 0.50412 | 0.29658 | . |
| Time_F9:Port_nameWeymouth | 0.47708 | 0.29516 |  |
| Time_F10:Port_nameWeymouth | 1.04772 | 0.29826 | *** |
| Time_F11:Port_nameWeymouth | 1.42817 | 0.3559 | *** |
| Port_namePlymouth:mean_hs | 0.81641 | 0.13656 | *** |
| Port_nameWest Mersea:mean_hs | 1.4908 | 0.24574 | *** |
| Port_nameWeymouth:mean_hs | 1.30149 | 0.17373 | *** |
| *Observations* | *13278* | | |
| *Signif. codes: 0 ‘***’ 0.001 ‘**’ 0.01 ‘*’ 0.05 ‘.’ 0.1 ‘ ’ 1* | | | |

**Table S2.** Coefficients for Success model Eqn. 2

| **Coefficients:** | **Estimates** | **Std. Error** | **Pr(>\|z\|)** |
| --- | --- | --- | --- |
| (Intercept) | 0.882048 | 0.111828 | *** |
| mean_hsr1 | -0.035235 | 0.010603 | *** |
| mean_hsr2 | -0.088608 | 0.029968 | ** |
| mean_hsr3 | -0.143398 | 0.169473 |  |
| mean_hsr4 | -0.663104 | 0.447051 |  |
| price_change | -0.013953 | 0.002884 | *** |
| vessel_id13 | -0.234831 | 0.146437 |  |
| vessel_id16 | -0.271115 | 0.115431 | * |
| vessel_id21 | -0.5056 | 0.12883 | *** |
| vessel_id43 | -0.117583 | 0.157501 |  |
| vessel_id47 | -0.417999 | 0.121167 | *** |
| vessel_id48 | 0.030674 | 0.151436 |  |
| vessel_id55 | -0.029619 | 0.151365 |  |
| vessel_id56 | -0.732218 | 0.145266 | *** |
| vessel_id59 | -0.44641 | 0.148045 | ** |
| vessel_id63 | -0.694191 | 0.174663 | *** |
| vessel_id68 | -0.133228 | 0.192855 |  |
| vessel_id69 | -0.838183 | 0.129204 | *** |
| vessel_id81 | -0.954925 | 0.143791 | *** |
| vessel_id89 | -0.443469 | 0.135121 | ** |
| vessel_id92 | -0.694151 | 0.151387 | *** |
| vessel_id93 | -0.829517 | 0.139788 | *** |
| vessel_id101 | 0.108991 | 0.135256 |  |
| vessel_id107 | 0.209084 | 0.120204 | . |
| vessel_id119 | -0.008595 | 0.117105 |  |
| vessel_id120 | -0.255232 | 0.166542 |  |
| vessel_id121 | 0.142423 | 0.11793 |  |
| vessel_id132 | -0.202831 | 0.138104 |  |
| vessel_id135 | -0.633855 | 0.146704 | *** |
| vessel_id136 | -0.199661 | 0.137845 |  |
| vessel_id160 | -1.260379 | 0.157964 | *** |
| vessel_id162 | -0.054863 | 0.128494 |  |
| vessel_id164 | -0.114629 | 0.121475 |  |
| vessel_id171 | 0.137812 | 0.116195 |  |
| vessel_id178 | -0.150051 | 0.125312 |  |
| vessel_id179 | -0.01622 | 0.119351 |  |
| vessel_id183 | -0.148641 | 0.117808 |  |
| vessel_id187 | -0.341761 | 0.11535 | ** |
| vessel_id194 | -0.802073 | 0.132871 | *** |
| vessel_id199 | -0.509809 | 0.128694 | *** |
| vessel_id211 | -0.167905 | 0.127416 |  |
| vessel_id212 | -0.425032 | 0.192823 | * |
| vessel_id215 | -0.7002 | 0.135829 | *** |
| vessel_id219 | -0.625107 | 0.185926 | *** |
| vessel_id222 | -0.695224 | 0.185925 | *** |
| vessel_id231 | -0.314065 | 0.174796 | . |
| vessel_id237 | -0.234348 | 0.116574 | * |
| vessel_id240 | -0.239747 | 0.118739 | * |
| vessel_id243 | -0.532558 | 0.146715 | *** |
| vessel_id260 | -0.252448 | 0.136841 | . |
| vessel_id265 | 0.131024 | 0.125388 |  |
| vessel_id278 | 0.051319 | 0.148712 |  |
| vessel_id282 | -0.543862 | 0.157448 | *** |
| vessel_id286 | -0.674815 | 0.11839 | *** |
| vessel_id290 | -0.073338 | 0.117577 |  |
| vessel_id291 | 0.23684 | 0.17483 |  |
| vessel_id292 | -0.123679 | 0.139664 |  |
| vessel_id298 | -0.113263 | 0.193056 |  |
| vessel_id302 | -0.275293 | 0.170517 |  |
| vessel_id306 | 0.349309 | 0.160319 | * |
| vessel_id310 | -0.567098 | 0.126361 | *** |
| vessel_id312 | -0.019767 | 0.13892 |  |
| vessel_id321 | -0.983429 | 0.163048 | *** |
| vessel_id325 | -0.094321 | 0.13125 |  |
| vessel_id328 | -0.354967 | 0.121113 | ** |
| vessel_id333 | 0.301551 | 0.12172 | * |
| vessel_id337 | 0.75773 | 0.179884 | *** |
| vessel_id339 | -0.259192 | 0.124293 | * |
| vessel_id341 | -0.291618 | 0.11924 | * |
| vessel_id342 | -0.22227 | 0.147914 |  |
| vessel_id345 | -0.10561 | 0.130407 |  |
| vessel_id346 | -0.559051 | 0.146604 | *** |
| vessel_id347 | -0.263862 | 0.170088 |  |
| vessel_id348 | -0.148419 | 0.134538 |  |
| vessel_id353 | -0.164866 | 0.122626 |  |
| vessel_id354 | -0.136339 | 0.118778 |  |
| vessel_id358 | -0.49057 | 0.12889 | *** |
| vessel_id360 | -0.002579 | 0.142594 |  |
| vessel_id361 | 0.038115 | 0.151151 |  |
| vessel_id364 | 0.371916 | 0.166827 | * |
| vessel_id369 | -0.686104 | 0.119703 | *** |
| vessel_id371 | -0.288773 | 0.180844 |  |
| vessel_id372 | 0.093003 | 0.18618 |  |
| vessel_id377 | -0.151009 | 0.117908 |  |
| vessel_id378 | -0.444397 | 0.142968 | ** |
| vessel_id379 | -0.454061 | 0.126557 | *** |
| vessel_id387 | -0.575262 | 0.117643 | *** |
| vessel_id388 | 0.12595 | 0.125412 |  |
| vessel_id389 | -0.182025 | 0.118785 |  |
| vessel_id390 | 0.209047 | 0.11406 | . |
| vessel_id391 | -0.393117 | 0.128177 | ** |
| vessel_id392 | -0.390957 | 0.133125 | ** |
| vessel_id393 | -0.854372 | 0.162977 | *** |
| vessel_id394 | -0.193814 | 0.117747 | . |
| vessel_id400 | -0.998976 | 0.137153 | *** |
| vessel_id408 | -0.016717 | 0.140533 |  |
| vessel_id410 | 0.054023 | 0.119785 |  |
| vessel_id411 | -0.282638 | 0.115656 | * |
| vessel_id412 | -0.615609 | 0.116564 | *** |
| vessel_id414 | -0.42587 | 0.11921 | *** |
| vessel_id415 | -0.354622 | 0.132199 | ** |
| vessel_id416 | -0.303555 | 0.142665 | * |
| vessel_id418 | -0.099567 | 0.119396 |  |
| vessel_id419 | -0.619474 | 0.167004 | *** |
| vessel_id420 | -0.400642 | 0.163519 | * |
| vessel_id422 | 0.121783 | 0.119123 |  |
| vessel_id423 | 0.209912 | 0.115359 | . |
| vessel_id427 | -0.260952 | 0.120921 | * |
| vessel_id428 | 0.003511 | 0.131043 |  |
| vessel_id430 | -0.774729 | 0.193081 | *** |
| vessel_id431 | 0.077003 | 0.151233 |  |
| vessel_id434 | -0.567203 | 0.133328 | *** |
| vessel_id435 | -0.325312 | 0.135532 | * |
| vessel_id438 | -0.393286 | 0.174895 | * |
| vessel_id443 | -0.227639 | 0.127678 | . |
| vessel_id445 | -0.271582 | 0.151641 | . |
| vessel_id448 | -0.386843 | 0.119577 | ** |
| vessel_id449 | -0.144921 | 0.114013 |  |
| vessel_id451 | 0.179578 | 0.115844 |  |
| vessel_id454 | -0.172454 | 0.160786 |  |
| vessel_id457 | -0.627976 | 0.123113 | *** |
| vessel_id458 | -0.716978 | 0.146504 | *** |
| vessel_id460 | -0.361209 | 0.135082 | ** |
| vessel_id461 | -0.484931 | 0.185651 | ** |
| vessel_id464 | 0.197084 | 0.160108 |  |
| vessel_id466 | 0.016801 | 0.119696 |  |
| vessel_id467 | -0.395992 | 0.130443 | ** |
| vessel_id469 | -0.163483 | 0.115616 |  |
| vessel_id470 | -0.368841 | 0.148173 | * |
| vessel_id473 | -0.773556 | 0.122655 | *** |
| vessel_id478 | 0.100286 | 0.116365 |  |
| vessel_id479 | -0.150223 | 0.130906 |  |
| vessel_id481 | -0.28821 | 0.170467 | . |
| vessel_id483 | -0.86852 | 0.166861 | *** |
| vessel_id487 | 0.20876 | 0.179988 |  |
| vessel_id488 | -0.30349 | 0.12186 | * |
| vessel_id493 | -0.275667 | 0.123874 | * |
| vessel_id494 | -0.255734 | 0.129301 | * |
| vessel_id495 | -0.220427 | 0.127103 | . |
| vessel_id497 | -0.17912 | 0.126244 |  |
| vessel_id498 | 0.140055 | 0.193457 |  |
| vessel_id499 | -0.022843 | 0.124846 |  |
| vessel_id500 | -0.155012 | 0.139429 |  |
| vessel_id504 | -0.258477 | 0.151908 | . |
| year2015 | -0.037601 | 0.015736 | * |
| year2016 | -0.105457 | 0.01594 | *** |
| year2017 | -0.129264 | 0.016813 | *** |
| year2018 | -0.033632 | 0.017329 | . |
| *Observations* | 8815 | | |
| *Signif. codes: 0 ‘***’ 0.001 ‘**’ 0.01 ‘*’ 0.05 ‘.’ 0.1 ‘ ’ 1* | | | |


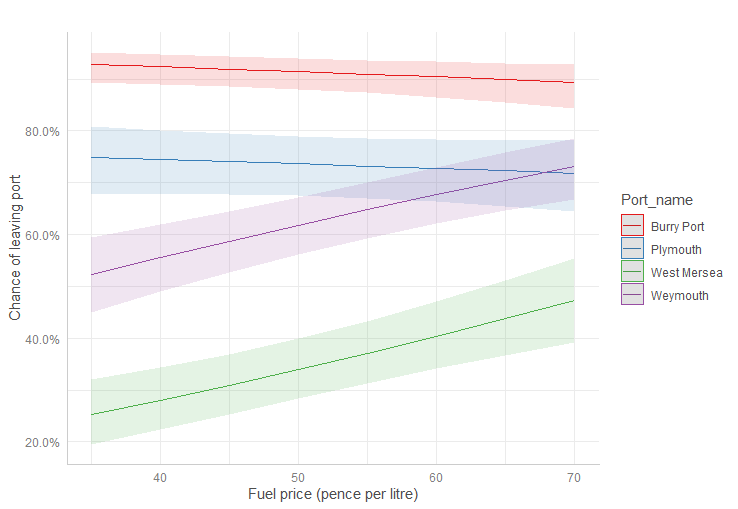
Fig. S1 Prediction of whether a vessel will leave port with changes to fuel price (pence per litre of red diesel) from the binary logistic regression (Eqn. 1 + fuel price as a predictor).


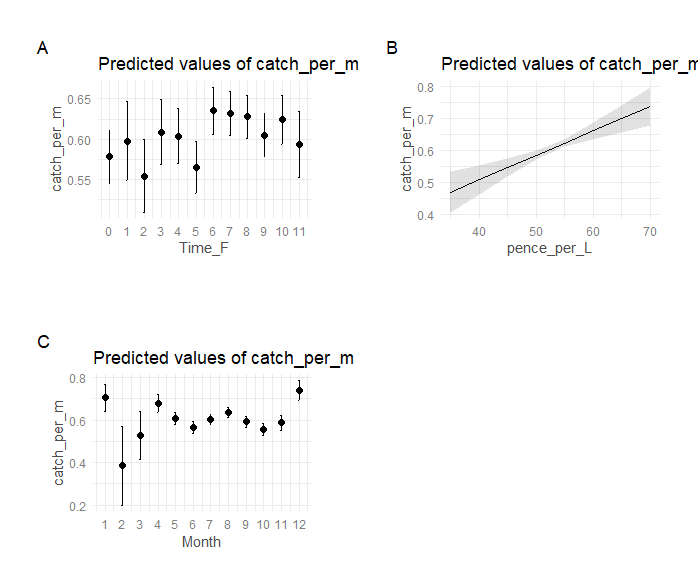
Fig. S2 Predictors of fishing success, measured as landed weight per metre of vessel length, from the regression analysis (Eqn. 2 + time of high tide + fuel price and Month of fishing trip); A) time of high tide, B) fuel price, C) Month the fishing trip took place
